# Exploring EGFR, Nectin-4, and TROP-2 as Therapeutic Targets for Bladder Cancer Photoimmunotherapy

**DOI:** 10.3390/molecules30244802

**Published:** 2025-12-17

**Authors:** Isis Wolf, Nora Giess, Céline Roider, Susanne Schultze-Seemann, Jonas Storz, Daniel B. Werz, Arkadiusz Miernik, Christian Gratzke, Philipp Wolf

**Affiliations:** 1 Department of Urology, Medical Center-University of Freiburg, Hugstetter Str. 55, 79106 Freiburg, Germany; isis.wolf@uniklinik-freiburg.de (I.W.); nora.giess@uniklinik-freiburg.de (N.G.); arkadiusz.miernik@uniklinik-freiburg.de (A.M.); christian.gratzke@uniklinik-freiburg.de (C.G.); 2 Faculty of Medicine, University of Freiburg, 79106 Freiburg, Germany; 3 Institute of Organic Chemistry, Albert-Ludwigs-Universität Freiburg, Albertstr. 21, 79104 Freiburg, Germany; jonas.storz@ocbc.uni-freiburg.de (J.S.); daniel.werz@chemie.uni-freiburg.de (D.B.W.)

**Keywords:** EGFR, Nectin-4, TROP-2, photoimmunotherapy, antibodies, photosensitizer dye, WB692-CB2, bladder cancer

## Abstract

Background: Non-muscle invasive bladder cancer (NMIBC) has limited therapeutic options and high recurrence rates. Photoimmunotherapy (PIT) enables targeted tumor ablation using antibody-photosensitizer conjugates and light activation. We evaluated EGFR, Nectin-4, and TROP-2 as PIT targets using cysteine-modified antibodies conjugated to the photosensitizer WB692-CB2. Methods: Antibodies derived from Cetuximab (Cmb, anti-EGFR), Enfortumab (Enf, anti-Nectin-4), and Sacituzumab (Sac, anti-TROP-2) were engineered with T120C and D265C mutations in the heavy chains for site-specific dye conjugation. Binding of the conjugates to BC cells was tested by flow cytometry and light-induced cytotoxicity of the conjugates, alone or in combination, was assessed by viability assays following irradiation. Results: Cysteine-modified antibodies were produced as intact IgG molecules and were efficiently conjugated with WB692-CB2 without loss of antigen specificity. Sac^T120C/D265C^-WB692-CB2 showed the highest target binding and achieved near-complete cell killing at a red-light dose of 32 J/cm^2^. Cmb^T120C/D265C^-WB692-CB2 required a fourfold higher light dose for comparable efficacy, while Enf^T120C/D265C^-WB692-CB2 demonstrated lower potency. No cytotoxicity was observed in antigen-negative cells. Combined treatment enhanced cytotoxicity, indicating additive phototherapeutic effects. Conclusions: Our findings suggest that PIT targeting EGFR, Nectin-4, or TROP-2 merits further preclinical development as a targeted therapeutic approach for NMIBC, including potential combinatorial or personalized strategies.

## 1. Introduction

Bladder cancer (BC) ranks among the ten most common cancers globally, with over 570,000 individuals newly diagnosed each year and around 210,000 deaths attributed to the disease. The highest incidence rates are observed in Southern Europe (with age-standardized incidence rates of 26.6 for males and of 5.8 for females per 100,000) [1].

At the time of detection, about 70% of tumors are restricted to the urothelial layer or the underlying lamina propria and have not invaded the muscle layer [2]. For non-muscle-invasive BC (NMIBC), transurethral resection followed by intravesical chemotherapy with mitomycin C or gemcitabine is the standard therapy. Patients with intermediate, high, and very high risk may also receive Bacillus Calmette-Guérin (BCG) immunotherapy to reduce the likelihood of disease progression [3]. However, recurrence rates remain high, affecting approximately 31–78% of patients within 5 years after treatment depending on the risk group [4]. Regular cystoscopic follow-up is therefore required. Progression to muscle-invasive BC (MIBC) necessitates radical treatment such as cystectomy with chemotherapy or radio-chemotherapy [5]. In metastatic disease, prognosis is poor, with median survival of only 2–3 months [6,7]. Recent advances in the treatment of BC include the use of immune checkpoint inhibitors (ICIs) [8] and antibody-drug conjugates (ADCs) such as enfortumab vedotin [9] and sacituzumab govitecan [10]. Despite these developments, clinical outcomes remain unsatisfactory. Novel targeted therapies are needed to eradicate residual tumor cells in localized disease, reduce recurrence, and improve both survival and quality of life.

Photoimmunotherapy (PIT) represents a highly specific approach within the field of targeted photodynamic therapy (PDT). In this strategy, tumor-specific monoclonal antibodies, antibody fragments, or ligands are covalently coupled to photosensitizers (PSs), enabling preferential accumulation of the conjugates in malignant tissues through recognition of cell-surface antigens. Once localized in the tumor, the PS moiety can be selectively activated by radiation with red or near-infrared light, which penetrates tissue effectively and is non-ionizing. When the PS is activated, photochemical reactions generate various reactive oxygen species (ROS), such as singlet oxygen, peroxides, and hydroxyl radicals. ROS induce oxidative stress, leading to damage to cellular organelles and biomolecules, followed by the release of neoantigens and damage-associated molecular patterns (DAMPs). These molecules stimulate pattern recognition receptors (PRRs) on dendritic cells and other antigen-presenting cells, driving their maturation and the activation of CD4^+^ and CD8^+^ T cells. This initiates and amplifies an adaptive anti-tumor immune response, including promotion of pro-inflammatory M1 macrophage polarization and reduced Treg-mediated immunosuppression. Overall, DAMP release during immunogenic cell death shifts the tumor microenvironment toward effective anti-tumor immunity [11,12]. PIT is particularly suitable for tumors that both (i) express antigens amenable to antibody targeting and (ii) are accessible to light delivery, either externally or via interstitial fiber-optic approaches. Compared with conventional chemotherapy or radiotherapy, PIT offers spatiotemporal precision and reduced systemic toxicity while retaining the immunological benefits of PDT [13].

For the PIT of NMIBC, the cell surface antigens epidermal growth factor receptor (EGFR), Nectin cell adhesion molecule 4 (Nectin-4), and Trophoblast cell surface antigen-2 (TROP-2), amongst others, are conceivable targets because they contribute to tumorigenesis and are overexpressed in BC. Moreover, antibody binding triggers their internalization, which can be exploited in PIT to enhance intracellular accumulation of the conjugates in the tumor cells. Overexpression of EGFR, a tyrosine kinase of the ErbB receptor family, correlates with tumor grade, muscle invasion, and recurrence of BC, and therefore serves as a prognostic marker. Based on immunohistological studies, EGFR is present in about 12–40% of NMIBC patients and undergoes internalization upon binding of anti-EGFR antibodies such as Cetuximab [14,15,16].

The cell adhesion molecule Nectin-4 is markedly overexpressed in various solid tumors, including urothelial cancer, and can be used to transport antibody-based therapeutics into the target cells by internalization [17,18,19]. It promotes tumor angiogenesis, cell growth, proliferation, migration, and epithelial–mesenchymal transition (EMT) and has been reported to be heterogeneously expressed in approximately 87–90% of patients with NMIBC [20,21,22]. Nectin-4 is the target of the ADC enfortumab vedotin (EV), which is indicated as first-line therapy in combination with pembrolizumab for patients who are ineligible for platinum chemotherapy. It is also used in patients with locally advanced or metastatic urothelial carcinoma who have already been treated with an ICI and a platinum-containing regimen [23].

TROP-2 is a type I transmembrane glycoprotein consisting of 323 amino acids and was found to be internalized after antibody binding [24]. It functions as a calcium signal transducer and contributes to proliferation, adhesion, EMT, and tumor cell death [17,25,26]. In an immunohistological panel of 102 patients with NMIBC, TROP-2 expression was detected in all samples and high expression of TROP-2 was significantly associated with tumor grade, stage, and recurrence [27]. TROP-2 is the antigen targeted by the antibody–drug conjugate sacituzumab govitecan. This therapy is approved for patients with locally advanced or metastatic urothelial carcinoma who have already undergone treatment with both platinum-based chemotherapy and an ICI [28].

We have recently published the novel silicon phthalocyanine dye WB692-CB2 (Abs._max_ 692 nm/Em._max_ 703 nm), the first light-activatable PS that can be directly conjugated to cysteine residues via a maleimide linker. Following incubation of prostate cancer cells with a conjugate composed of a cysteine-modified antibody targeting the prostate-specific membrane antigen (PSMA) and WB692-CB2, subsequent red-light irradiation effectively induced pyroptosis as the predominant mode of cell death [29]. In the present study, we established a PIT strategy against BC cells employing WB692-CB2-conjugated antibodies directed against EGFR, Nectin-4, and TROP-2.

## 2. Results

**Figure 1:**
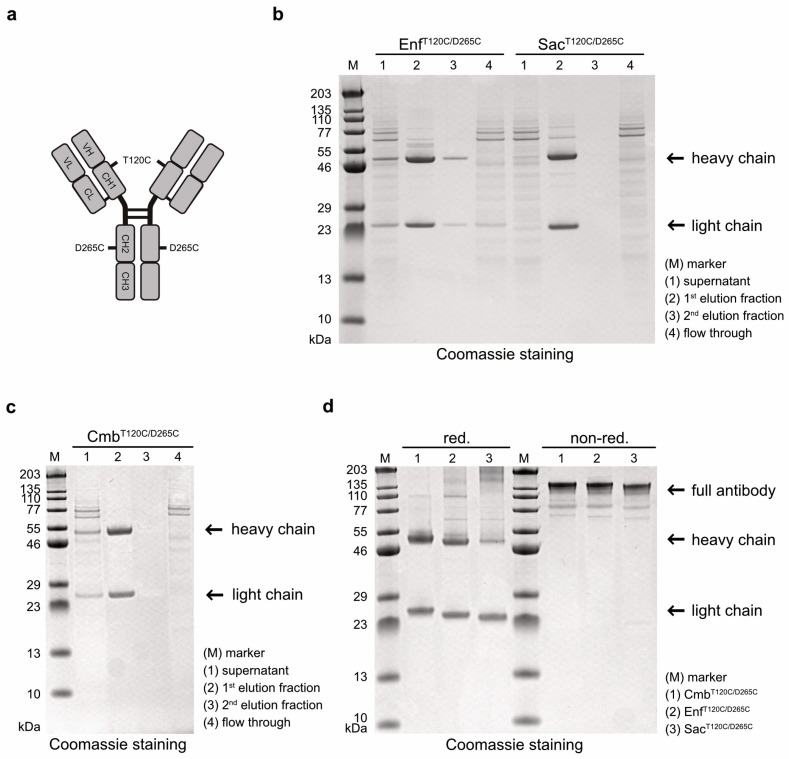
(**a**) Graphical representation of the antibodies carrying cysteine mutations T120C and D265C in the heavy chains, enabling coupling of the photosensitizer dye WB692-CB2. (**b**) SDS-PAGE analysis of the antibodies Enf^T120C/D265C^ and Sac^T120C/D265C^ after purification from cell culture supernatants by Protein G affinity chromatography. (**c**) SDS-PAGE analysis of the antibody Cmb^T120C/D265C^ after purification from cell culture supernatant by Protein G affinity chromatography. (**d**) SDS-PAGE of the antibodies after purification under reducing and non-reducing conditions.

Antibody variants targeting EGFR, Nectin-4, and TROP-2 were generated by cloning the VH and VL domains of Cetuximab (Cmb), Enfortumab (Enf) and Sacituzumab (Sac), respectively, into human hIgG1 heavy- and light-chain expression vectors carrying cysteine mutations at positions T120 and D265 in the heavy chain (Figure 1a). After expression in Expi293T cells and purification by affinity chromatography, the cysteine-modified antibodies Cmb^T120C/D265C^, Enf^T120C/D265C^, and Sac^T120T/D265C^ were analyzed by SDS-PAGE. All antibodies were detected in the first elution fraction (Figure 1b,c). Under reducing conditions, the expected ~49–50 kDa heavy chains and ~23 kDa light chains were observed, whereas under non-reducing conditions, intact antibodies with a molecular weight of ~145 kDa were detected (Figure 1d). This proved that all antibody variants were correctly assembled as full-length IgG molecules in our expression system.

**Figure 2:**
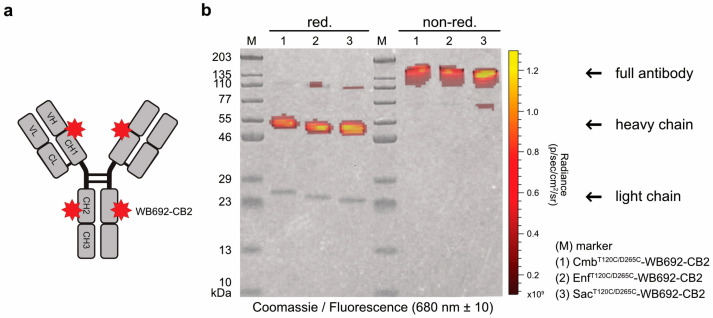
(**a**) Graphical representation of the antibody-dye conjugates carrying cysteine-coupled WB692-CB2 dyes. (**b**) Overlay of Coomassie-stained SDS-PAGE gels of the conjugates under reducing and non-reducing conditions, visualized under white and red light (680 ± 10 nm).

In the next step, the PS dye WB692-CB2 was coupled to the cysteine-modified antibodies via a maleimide linker (Figure 2a). The introduction of two cysteine mutations per heavy chain theoretically allows the coupling of up to four dye molecules per antibody. Using our established coupling protocol, in which ten dye molecules were added per antibody molecule, we achieved dye-to-antibody ratios of 3.05, 3.08, and 3.13 for Cmb^T120C/D265C^-WB692-CB2, Enf^T120C/D265C^-WB692-CB2, and Sac^T120C/265C^-WB692-CB2, respectively. All three conjugates were generated in a single coupling reaction. The narrow range of the dye-to-antibody ratios indicated that the maleimide–thiol coupling reaction proceeded with comparable efficiency across the three different antibody variants, demonstrating the robustness and reproducibility of the conjugation protocol. An analysis of the conjugates under red light (680 ± 10 nm) revealed the calculated molecular masses of 152.6 kDa for Cmb^T120C/D265C^-WB692-CB2, 151.3 kDa for Enf^T120C/D265C^-WB692-CB2, and 152.8 kDa for Sac^T120C/D265C^-WB692-CB2 in agreement with the observed shift in apparent molecular weight on SDS-PAGE in almost complete absence of major degradation products or aggregates. Fluorescence signals corresponding to the heavy chains confirmed successful dye coupling to the cysteine mutations (Figure 2b). To exclude structural alterations arising from the reduction and re-oxidation steps, we subsequently verified that the antigen-binding properties of the antibody-dye conjugates remained intact.

**Figure 3:**
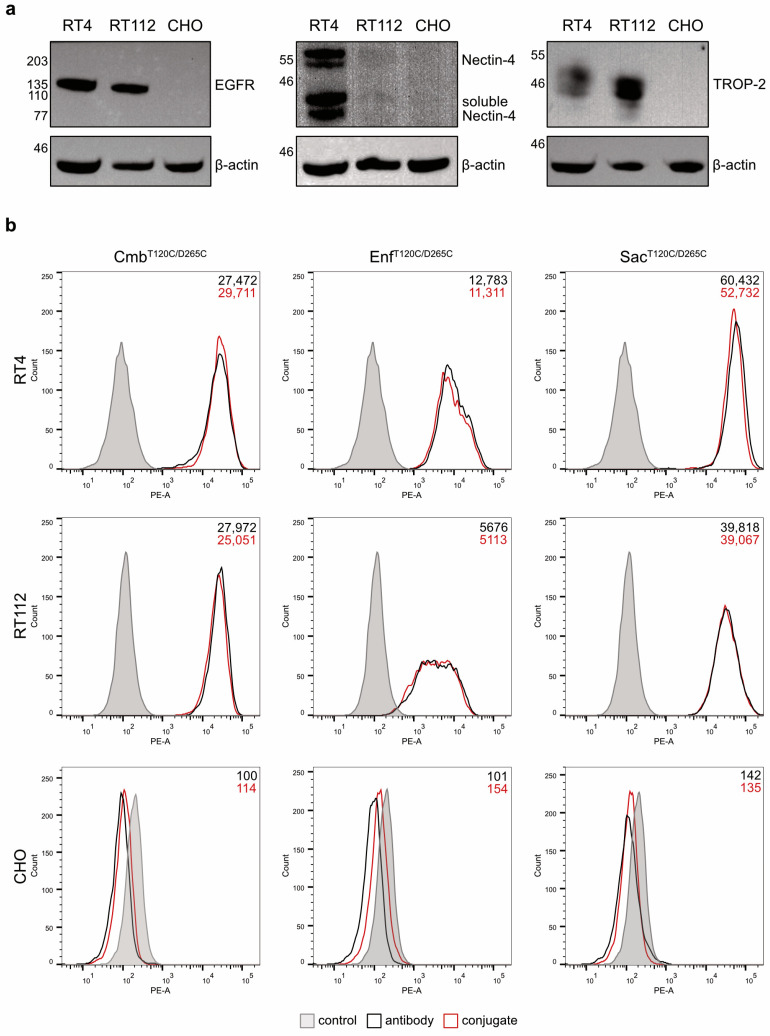
(**a**) Western blot analysis of EGFR, Nectin-4, and TROP-2 expression in the BC cell lines RT4 and RT112 and in the antigen-negative control cell line CHO. (**b**) Cell binding of the antibodies and conjugates to the target cells as demonstrated by flow cytometry. Binding of the isotype control antibody is shown in gray.

The binding properties of the antibodies and their conjugates were evaluated on RT4 and RT112 BC cells. Western blot analyses of cell lysates confirmed that RT4 cells were EGFR^high^, Nectin-4^high^ and TROP-2^high^, whereas RT112 cells were characterized as EGFR^high^, Nectin-4^low^ and TROP-2^high^. The hamster cell line CHO served as a negative control and showed no detectable expression of any of the three antigens (Figure 3a). Flow cytometry revealed strong and specific binding of the antibodies and their corresponding conjugates to RT4 and RT112 cells, with the highest mean fluorescence intensity (MFI) values observed for Sac^T120C/D265C^, followed by Cmb^T120C/D265C^ and Enf^T120C/D265C^, and their respective conjugates. No binding was detected in CHO cells (Figure 3b). These results proved that the coupling of the WB692-CB2 dye did not impair antigen-specific binding of the antibodies.

For PIT, BC cells were incubated with 10 µg/mL of each conjugate and subsequently irradiated with varying doses of red light (690 nm, 0–128 J/cm^2^). All conjugates induced light dose-dependent cytotoxicity in the target cells (Sac^T120C/D265C^-WB692-CB2 > Cmb^T120C/D265C^-WB692-CB2 > Enf^T120C/D265C^-WB692-CB2), consistent with their relative binding affinities. The exception was Enf^T120C/D265C^-WB692-CB2 with an apparent cytotoxicity plateau in RT112 cells, above which higher irradiation doses provided only minimal additional effects.

**Figure 4:**
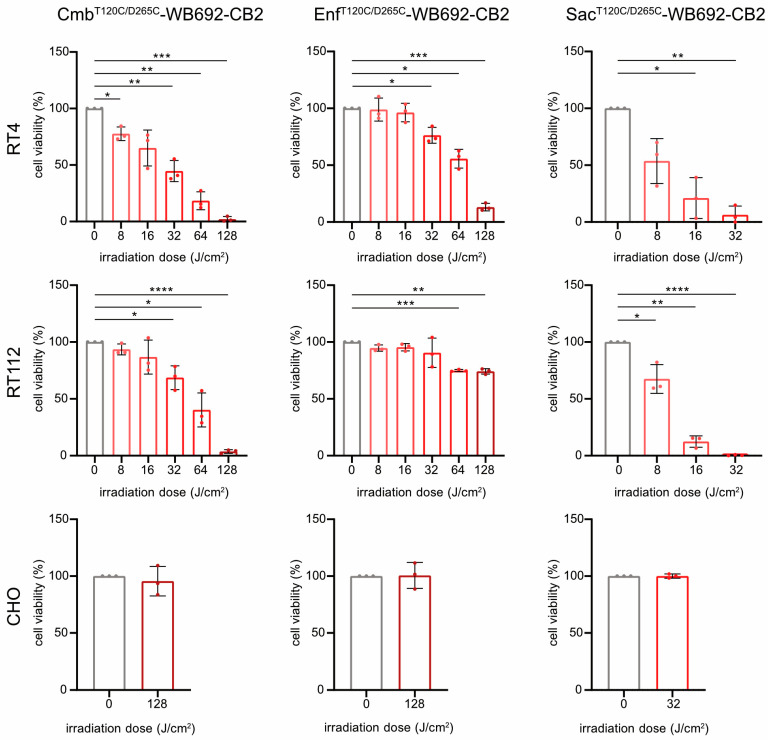
Cytotoxicity of the conjugates Cmb^T120C/D265C^-WB692-CB2, Enf^T120C/D265C^-WB692-CB2, and Sac^T120C/D265C^-WB692-CB2 after irradiation with different light doses in RT4 and RT112 cells. CHO cells served as antigen-negative controls. Mean values ± SD of three independent biological experiments. Statistical analyses were performed using unpaired, parametric Student’s *t*-tests with Welch’s correction (* *p* < 0.05, ** *p* < 0.01, *** *p* < 0.005, **** *p* < 0.001).

Treatment with Sac^T120C/D265C^-WB692-CB2 significantly reduced RT4 cell viability to 6.3 ± 7.6% and completely eradicated RT112 cells at a light dose of 32 J/cm^2^. Nearly complete ablation of BC cells was achieved with Cmb^T120C/D265C^-WB692-CB2 at a dose of 128 J/cm^2^, resulting in 2.1 ± 2.3% and 3.6 ± 1.5% viability for RT4 and RT112 cells, respectively. In contrast, Enf^T120C/D265C^-WB692-CB2 exhibited the weakest phototoxic response, reducing viability of RT4 cells with Nectin-4^high^ expression to 13.1 ± 3.3% and viability of RT112 cells with Nectin-4^low^ expression to only 74.2 ± 2.4% at 128 J/cm^2^. No cytotoxic effects were observed in the antigen-negative CHO control under any conditions, demonstrating the high specificity and safety of our PIT approach (Figure 4). Microscopic analysis of the cells after photoimmunotherapy revealed that alterations in viability was indicative of cell death and not of growth arrest (Supplementary Figure S1).

**Figure 5:**
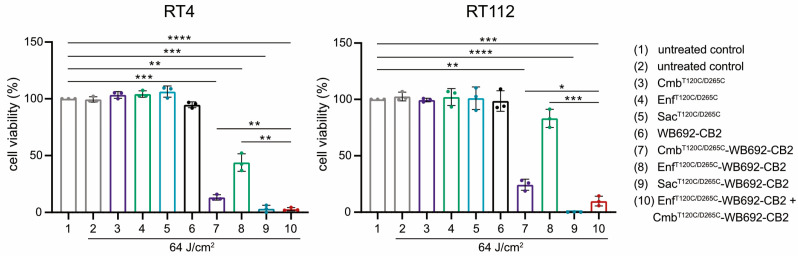
Cytotoxicity of the conjugates Cmb^T120C/D265C^-WB692-CB2, Enf^T120C/D265C^-WB692-CB2 and Sac^120T/265C^-WB692-CB2 alone and in combination after irradiation with a light dose of 64 J/cm^2^ in RT4 and RT112 cells. Control samples were treated with the uncoupled antibodies or the free dye. Mean values ± SD of three independent biological experiments. Statistical analyses were performed using unpaired, parametric Student’s *t*-tests with Welch’s correction (* *p* < 0.05, ** *p* < 0.01, *** *p* < 0.005, **** *p* < 0.001).

The simultaneous application of multiple conjugates was investigated to assess potential additive cytotoxic effects. BC cells were co-incubated with both Cmb^T120C/D265C^-WB692-CB2 and Enf^T120C/D265C^-WB692-CB2 simultaneously and subsequently irradiated with a light dose of 64 J/cm^2^. Indeed, the combined treatment resulted in a markedly enhanced cytotoxic response compared with either conjugate alone, indicating additive phototoxic effects. In contrast, neither the unconjugated antibodies nor the free WB692-CB2 dye exhibited any cytotoxicity under the same experimental conditions, confirming that light-induced cell killing was strictly dependent on targeted photoactivation of the conjugates (Figure 5).

Collectively, these results demonstrate that cysteine-engineered antibody–dye conjugates targeting EGFR, Nectin-4, and TROP-2 retain their antigen specificity and induce highly selective, light-dependent cytotoxicity in BC cells. Moreover, the additive effects observed upon combined application of different conjugates suggest an additive therapeutic potential that could enhance treatment efficacy and offer meaningful benefits for patients with heterogeneous tumor antigen expression.

## 3. Discussion

EGFR, Nectin-4, and TROP-2 represent valuable targets for the PIT of NMIBC, as they are highly expressed on the surface of BC cells and undergo internalization after antibody binding [15,18,26]. In this study, we demonstrate that the antigen-binding domains of Cetuximab, Enfortumab, and Sacituzumab—originally employed as ADCs for the targeted delivery of cytotoxic agents to cancer cells [30,31]—can also be used to generate cysteine-modified antibodies as vehicles for intracellular transport of the photosensitizer dye WB692-CB2 in PIT. Consequently, PIT against BC could be extended to other therapeutic antibodies currently being evaluated in clinical trials, e.g., ACDs directed against human epidermal growth factor receptor 2 (HER-2), tissue factor (TF), or epithelial cell adhesion molecule (EpCAM) [32].

Based on the MFI values from our flow cytometric analyses, we observed the highest binding of the anti-TROP-2 conjugate Sac^T120C/D265C^-WB692-CB2 to RT4 and RT112 cells, followed by the anti-EGFR conjugate Cmb^T120C/D265C^-WB692-CB2 and the anti-Nectin-4 conjugate Enf^T120C/D265C^-WB692-CB2. Accordingly, the highest cytotoxicity after PIT was achieved with Sac^T120C/D265C^-WB692-CB2. At a conjugate concentration of 10 µg/mL and a light dose of 32 J/cm^2^, nearly complete elimination of the BC cells was observed. In contrast, Cmb^T120C/D265C^-WB692-CB2 required a fourfold higher light intensity (128 J/cm^2^) to achieve comparable efficacy, while Enf^T120C/D265C^-WB692-CB2 induced only limited cytotoxicity, reducing RT112 and RT4 cell viability by approximately 26% and 87% at the same dose, respectively. Overall, we identified Sac^T12C0/D265C^-WB692-CB2 as the most effective conjugate for PIT of BC cells, followed by Cmb^T120C/D265C^-WB692-CB2 and Enf^T120C/D265C^-WB692-CB2. Beyond differences in antigen binding, the observed variation in cytotoxicity may be attributable to differences in internalization kinetics of the conjugates. For example, RS7, the murine antibody variant of Sacituzumab, was found to be rapidly internalized into breast cancer cells with approximately 50% of antibody internalized within 70 min [33]. In contrast, only weak internalization was observed for Cetuximab in different cancer cells within 24 h [34]. Moreover, the efficacy of PIT might be influenced by differences in endolysosomal trafficking of the conjugate, resulting in varying degrees of inactivation of the PS in the lysosomes over time. At the same time, however, light exposure might cause the PS in the endolysosomes to induce cell stress and damage the endolysosomal membranes, leading to their rupture. As a result, lysosomal enzymes are released into the cytosol, disrupting cellular homeostasis and initiating cell death. Further investigations will be necessary to clarify the relative contributions of these processes to PIT efficacy with our dye conjugates.

Our observations are in line with other studies. Railkar and colleagues used a conjugate consisting of the anti-EGFR antibody panitumumab and the lysine-coupled PS dye IR700 for the PIT of BC. The conjugate showed the best efficacy against bladder squamous cells carcinoma cells with the highest EGFR expression and significantly reduced tumor growth in a BC mouse xenograft model [35]. In a recent study, a conjugate consisting of an anti-Nectin 4 antibody (clone Enfbio) and IR700 effectively killed luminal bladder cancer cells in vitro, suppressed tumor growth, improved survival in multiple xenograft models, and enabled tumor imaging, demonstrating strong potential as a treatment for luminal subtype bladder cancer [36].

We recently showed that PIT with WB692-CB2 induced pyroptosis as the main cell death mechanism in prostate cancer cells [29]. Pyroptosis is a non-apoptotic form of programmed cell death characterized by inflammasome activation and caspase-dependent cleavage of gasdermin D, resulting in the formation of pores in the cell membrane and subsequent cell burst [37]. This causes the release of pro-inflammatory molecules that recruit lymphocytes and boost the immune system’s capacity to eliminate tumor cells. Induction of pyroptosis by antineoplastic agents is increasingly coming into focus to combat apoptosis-resistant tumor cells and to enhance the efficacy of ICI [38]. Our future investigations will determine whether similar mechanisms occur in BC cells. If confirmed, PIT could not only eradicate tumor cells but also prime the immune system of NMIBC patients against tumor cells that have already spread [39]. The possible mechanism of PIT-induced pyroptosis, while advantageous for immunogenic cell death, could, however, also provoke substantial local inflammation in the confined bladder environment. This may exacerbate edema, pain, and mucosal damage, and in severe cases impair bladder function. In a clinical setting, PIT must therefore be carried out according to a careful treatment regimen in terms of conjugate dose and light intensity in order to avoid such side effects.

BC can be multifocal and exhibits pronounced intra- and intertumoral antigen heterogeneity, including variability in EGFR, Nectin-4 and TROP-2 expression, driven by genetic mutations, altered molecular pathways, and influences of the tumor microenvironment [22,40,41]. This inherent heterogeneity affects disease progression, recurrence, and therapeutic outcome [22,42,43]. Although PIT of NMIBC is not yet established in clinical practice, potential risks cannot be excluded due to heterogeneous, intra-, and intertumoral expression of the target antigens. Low antigen expression across tumor cell populations in NMIBC may lead to reduced binding and cellular accumulation of the conjugates, incomplete tumor eradication by PIT, and survival of resistant clones. In our study, we found that Enf^T120C/D265C^-WB692-CB2 showed no enhanced cytotoxicity in RT112 cells despite increasing the light dose. This could be due to the low Nectin-4 expression in this cell line, which could be the rate-limiting factor for the conjugate uptake. Our data based on Western blot and flow cytometric analyses represent semi-quantitative assessments of antigen expression levels and cannot clarify the threshold of sufficient antigen expression for effective PIT. However, whereas antigen expression is a rate-limiting factor for ADC uptake, payload delivery, and maximal efficacy, it is not necessarily a limiting factor for PIT because the induction of immunogenic cell death by PIT allows bystander immune effects that may compensate for incomplete antigen coverage. The light dose of 64 J/cm^2^ we used in our in vitro combination experiments is considered as a medium dose in animal models, where effective light doses vary between 50 J/cm^2^ to 100 J/cm^2^ depending, e.g., on the immune status of the animals, tumor model, kind of light source, antibody dye conjugate, or penetration depth [44,45].

Target-antigen expression, e.g., EGFR expression, on normal urothelial or peri-tumoral cells raises the possibility of off-tumor conjugate binding, resulting in unintended tissue injury when PIT triggers cytotoxicity. On the other site, high antigen density on tumor cells coupled with low expression on normal tissues enhances tumor selectivity, broadens the therapeutic window, and minimizes off-target effects.

For the treatment of tumors with variable antigen expression by PIT, it might be beneficial to target multiple tumor antigens sequentially or simultaneously to broaden tumor coverage. In our study, cotreatment of the BC cells with Cmb^T120C/D265C^-WB692-CB2 and Enf^T120C/D265C^-WB692-CB2 yielded additive cytotoxic effects. Improved cytotoxicity was also reached in a former PIT approach with an anti-EGFR panitumumab-IR700 conjugate in combination with an anti-HER-2 trastuzumab-IR700 conjugate compared to the corresponding monotherapies in BC cells [46]. In the context of personalized medicine, antigen expression profiles could be assessed before and during PIT using biopsies enabling tailored administration of appropriate antibody–dye conjugates. This approach could improve therapeutic outcomes and reduce the risk of tumor recurrence.

**Figure 6:**
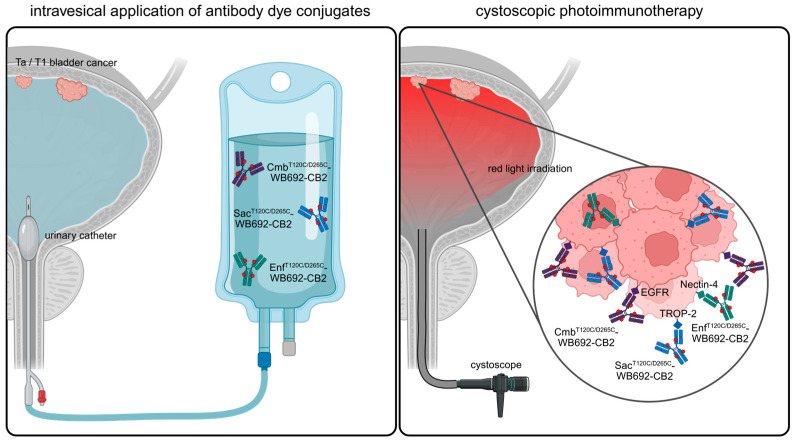
Graphical abstract illustrating the future clinical application of PIT using WB692-CB2 conjugates for the treatment of NMIBC. Following intravesical application of the antibody–dye conjugates into the bladder, they can be activated by cystoscopic red light for the selective eradication of BC cells expressing EGFR, Nectin-4 and/or TROP-2. For personalized PIT, a pre-therapeutic determination of target antigen expression in biopsy samples may guide the selection of an optimal conjugate combination. Created in BioRender. Wolf, I (2025) https://BioRender.com/sx866ld.

In this study, we demonstrated that PIT has the potential as a novel, targeted therapeutic option for NMIBC. In future clinical application, PIT could be performed by instilling the bladder with the respective antibody–dye conjugates via a catheter, followed by light irradiation using a cystoscope (Figure 6).

## 4. Materials and Methods

### 4.1. Cell Lines

The authenticated cell lines RT4, originating from a patient with a recurrent, well-differentiated papillary transitional tumor, and RT112, derived from a case of transitional cell carcinoma of the urinary bladder, both expressing EGFR, Nectin-4, and TROP-2, were obtained from the German Collection of Microorganisms and Cell Cultures (Leibniz Institute, Braunschweig, Germany). The CHO cell line, used as a negative control, was sourced from Gibco (Invitrogen, Karlsruhe, Germany). RT4 cells were cultivated in EMEM medium (Cytion, Eppelheim, Germany), RT112 cells in RPMI1640 medium (Gibco), and CHO cells in F-12 Nutrient Mixture Medium (Gibco) at 37 °C and 5% CO_2_. All culture media were enriched with 10% fetal calf serum (Sigma Aldrich, St. Louis, MO, USA) and 1% penicillin/streptomycin, respectively (100 U/mL, 100 mg/L, Sigma Aldrich).

### 4.2. Antibody Generation

For the generation of the heavy chains of the cysteine-modified antibodies Cetuximab (Cmb^T120C/D265C^, https://www.kegg.jp/entry/D03455, accessed on 30 March 2022)), Enfortumab (Enf^T120C/D265C^, https://www.kegg.jp/entry/D11524, accessed on 29 October 2024) and Sacituzumab (Sac^T120C/D265C^, https://www.kegg.jp/entry/D10984, accessed on 22 October 2024), the variable heavy-chain (VH) domains and constant heavy-chain (CH) domain genes of a hIgG1 antibody carrying the T120C and D265C (EU numbering) cysteine mutations were synthetized by Gene Art technology optimized for eukaryotic expression (Invitrogen, Regensburg, Germany). The variable light-chain (VL) domains of the three antibodies were synthesized in parallel. The heavy chains were cloned into the expression vector pCSEH1c and the light chains into the expression vector pCSL3k containing a human IgG1 constant light-chain (CL) domain, as described previously [29]. Constructs were transformed into XL1-Blue MRF’ supercompetent *E. coli* cells (Agilent Technologies, Waldbronn, Germany). Plasmid DNA was isolated using the NucleoBond^®^ Xtra Maxi Kit (Macherey-Nagel, Düren, Germany), and all constructs were sequence-confirmed by Microsynth Seqlab (Göttingen, Germany). 

Recombinant antibodies were subsequently produced in EXPI293F cells (Thermo Fisher Scientific, Waltham, MA, USA) using established protocols [29]. The antibodies were purified from cell culture supernatant by affinity chromatography. In short, the antibody-containing supernatant was diluted 1:1 with PBS (pH 7.0) and passed over a Protein G affinity column (Cytiva, Marlborough, MA, USA). Following column washing with PBS (pH 7.0), bound antibodies were eluted using 0.1 M glycine-HCl (pH 2.5) in two 1 mL fractions. The eluate was immediately neutralized with 1 M Tris-HCl (pH 9.0), dialyzed against PBS (pH 7.4), and stored at −20 °C. Antibody concentrations were finally measured with a NanoDrop Lite™ spectrophotometer (Thermo Fisher Scientific). 

### 4.3. Generation of the Antibody–Dye Conjugates

The cysteine-engineered antibodies Cmb^T120C/D265C^, Enf^T120C/D265C^ and Sac^T120C/D265C^ were first diluted in PBS containing 1 mM EDTA (pH 7.4) and then reduced with a 40-fold molar excess of TCEP (Tris-(2-carboxyethyl)phosphine hydrochloride; Carl Roth, Karlsruhe, Germany) for 3 h at 37 °C on a shaking platform. The reduced proteins were dialyzed overnight at 4 °C against PBS with 1 mM EDTA (pH 7.4), after which re-oxidation was carried out using dehydroascorbic acid (dhAA; Sigma-Aldrich, St. Louis, MO, USA) at a 30-fold molar excess for 4 h at room temperature. For fluorescent labeling, a 10-fold molar excess of WB692-CB2 dye was added and incubated for 1 h at room temperature while protected from light. The reaction was then quenched by adding N-acetyl-L-cysteine (25-fold molar excess) for 15 min. Unreacted dye was removed through Protein G affinity purification (Cytiva), followed by dialysis into PBS (pH 7.0). Final protein concentrations were measured using the Pierce BCA Protein Assay Kit (Thermo Fisher Scientific). The degree of labeling (dye-to-protein ratio) was calculated according to the Tech Tip #31 protocol “Calculate dyeprotein (F/P) molar ratios” (Thermo Fisher Scientific). To achieve this, the absorbance of the purified conjugate was determined spectrophotometrically by measuring at 280 nm (A_280_) and at the dye-specific absorption maximum of 690 nm (A_690_). The number of dye molecules per protein molecule was calculated as follows:

$$D/P=\frac{A_{690}\times \varepsilon_{Protein}}{\left(A_{280}-CF\times A_{690}\right)\times \varepsilon_{Dye}}$$

where CF is the correction factor accounting for dye absorbance at 280 nm (0.1582), ε_Dye_ is the molar extinction coefficient of the dye (116.000 M^−1^cm^−1^ in 1× PBS), and ε_Protein_ is the molar extinction coefficient of the antibody (210.000 M^−1^cm^−1^).

### 4.4. SDS PAGE 

The antibodies Cmb^T120C/D265C^, Enf^T120C/D265C^ and Sac^T120C/D265C^, as well as their corresponding antibody–dye conjugates Cmb^T120C/D265C^-WB692-CB2, Enf^T120C/D265C^-WB692-CB2 and Sac^T120C/D265C^-WB692-CB2, were analyzed by SDS-PAGE under reducing and non-reducing conditions. Protein bands were visualized by Coomassie staining (Protein Ark, Sheffield, UK) and fluorescence-based imaging (λ = 680 nm) using an IVIS 200 Imaging System (Revvity, Waltham, MA, USA). The theoretical molecular mass of the antibody–dye conjugates was calculated from the known molecular mass of the unconjugated antibodies (Cmb^T120C/D265C^ = 145.4 kDa, Enf^T120C/D265C^ = 144.1 kDa; Sac^T120C/D265C^ = 145.6 kDa) and the average number of about 3 molecules per antibody with 2.4 kDa each, yielding an estimated mass of 152.6 kDa for Cmb^T120C/D265C^-WB692-CB2, 151.3 kDa for Enf^T120C/D265C^-WB692-CB2, and 152.8 kDa for Sac^T120C/D265C^-WB692-CB2 in agreement with the observed shift in apparent molecular weight on SDS–PAGE.

### 4.5. Western Blot

Cells were lysed in a buffer composed of 50 mM Tris-HCl, 150 mM NaCl, 1 mM EDTA, 0.5% sodium deoxycholate, 0.05% SDS, and 1% Igepal. Protein levels were quantified using the Quick Bradford Protein Assay (Bio-Rad Laboratories, Hercules, CA, USA). Equivalent protein amounts (50 μg per lane) were loaded for SDS-PAGE and subsequently transferred to nitrocellulose membranes. Following transfer, EGFR, Nectin-4 and TROP-2 were detected using polyclonal rabbit anti-human EGFR IgG (#sc-03-G, Santa Cruz Biotechnology, Dallas, TX, USA), polyclonal rabbit anti-human Nectin-4 IgG (#17402S, Cell Signaling Technologies, Danvers, MA, USA), and monoclonal rabbit anti-human TROP-2 IgG (#90540, Cell Signaling Technologies). For secondary detection, an HRP-linked polyclonal goat anti-rabbit IgG antibody (#P0448, Dako Denmark A/S, Glostrup, Denmark) was applied. β-actin served as the loading control and was visualized using an HRP-conjugated mouse anti-human β-actin antibody (#HRP-66009, Proteintech Group Inc., Rosemont, IL, USA). Signals were visualized using an enhanced chemiluminescence (ECL) detection system and imaged with an INTAS Chemo Star Imager (INTAS Science Imaging Instruments, Göttingen, Germany).

### 4.6. Flow Cytometry 

Binding of the antibodies and their dye-labeled counterparts to EGFR-, Nectin-4-, and TROP-2-positive BC cell lines (RT112 and RT4) and to the antigen-negative control cell line CHO was analyzed by flow cytometry. To achieve this, 2 × 10^5^ cells/well were seeded and incubated with 10 µg/mL of each antibody or antibody dye conjugate in a dilution buffer containing PBS containing 3% fetal bovine serum, and 0.1% sodium azide for 1 h in the dark. A human IgG isotype control (Invitrogen, Karlsruhe, Germany) was used as negative control. After washing, cells were incubated with goat anti-human IgG-PE secondary antibody (Southern Biotech, Birmingham, AL, USA) (1:500) and eBioscience™ Fixable Viability Dye eFluor™ 450 (Invitrogen, Karlsruhe, Germany) (1:1000) for 30 min at 4 °C in the dark. After the final wash, cells were resuspended in dilution buffer and examined on a FACSymphony™ A1 Flow Cytometer (Becton Dickinson, Heidelberg, Germany). The resulting flow cytometry datasets were processed and evaluated using FlowJo™ v10 (Becton Dickinson). A standardized sequential gating strategy was applied to all samples. Initially, cell debris was removed by gating according to forward scatter area (FSC-A) and side scatter area (SSC-A). Singlet discrimination was performed using forward scatter high (FSC-H) versus FSC-A to remove doublets. Non-viable cells were excluded based on coloring by the Viability Dye. The final gate was applied to the PE fluorescence channel (PE-A) to determine the mean fluorescence (MFI) values.

### 4.7. Photoimmunotherapy

Target cells were plated at 1.25 × 10^5^ cells/mL in 35 mm culture dishes (Thermo Fisher Scientific, Waltham, MA, USA) and incubated for 24 h at 37 °C in a humidified atmosphere containing 5% CO_2_. On the following day, cells were treated for 24 h with either 10 µg/mL of the antibodies Cmb^T120C/D265C^, Enf^T120C/D265C^ or Sac^T120C/D265C^, their corresponding conjugates, or an equimolar concentration of free dye WB692-CB2. Untreated cells served as controls. After treatment, cells were rinsed three times with PBS, and phenol-red–free medium was added prior to illumination. Irradiation was carried out using a 690 nm light-emitting diode (LED L690-66-60, Marubeni, Tokyo, Japan) delivering an average intensity of 16 mW/cm^2^, with applied light doses ranging from 0 to 128 J/cm^2^. For combined PIT experiments, RT112 and RT4 cells were exposed to 10 μg/mL of either Cmb^T120C/D265C-^WB692-CB2 or Enf^T120C/D265C^-WB692-CB2 individually or in combination, followed by irradiation with 64 J/cm^2^ of red light.

Twenty-four hours after PIT, cells were detached using trypsin, stained with Erythrosin B (Logos Biosystems, Villeneuve d’Ascq, France), and viable cell numbers were quantified with a Neubauer counting chamber. The number of living cells of the untreated and non-irradiated control was defined as 100%, and numbers of living cells for the different treatment conditions were calculated relative to this control. Data are presented as mean ± SD from three independent experiments. Statistical comparisons were performed using an unpaired Student’s *t*-test with Welch’s correction in GraphPad Prism 7 (GraphPad Software, San Diego, CA, USA), with *p* < 0.05 regarded as statistically significant.

### 4.8. Microscopy

To analyze changes in cell morphology after PIT, cells were treated with the conjugates and irradiated with light. Cell damage was examined 24 h later using a Zeiss AxioObserver Z.1 inverted microscope (Carl Zeiss Microscopy GmbH, Munich, Germany).

## 5. Conclusions

Our in vitro study demonstrates that EGFR, Nectin-4, and TROP-2 are effective targets for the PIT of NMIBC using cysteine-engineered antibody-dye conjugates. Among them, the TROP-2-directed conjugate Sac^T120C/D265C^-WB692-CB2 showed the highest cytotoxic efficacy. Our findings provide the basis for further preclinical development of PIT for NMIBC, including investigations into cell death mechanisms and the in vivo efficacy of the conjugates. If successful, PIT could, in the future, be integrated into personalized, combinatorial treatment strategies for NMIBC to eliminate tumor cells and prevent recurrence. 

## Data Availability

The data presented in this study are available on request from the corresponding author.

## Supplementary Materials

The following supporting information can be downloaded at https://www.mdpi.com/article/10.3390/molecules30244802/s1, Figure S1: Morphological changes of RT4 and RT112 bladder cancer cells following PIT.

## Abbreviations

The following abbreviations are used in this manuscript:

ADC	Antibody–drug conjugate.
BC	Bladder cancer.
BCG	Bacillus Calmette–Guérin.
Cmb	Cetuximab.
DAMPs	Damage-associated molecular patterns.
EGFR	Epidermal growth factor receptor.
Enf	Enfortumab.
EpCAM	Epithelial cell adhesion molecule.
EV	Enfortumab Vedotin.
HER-2	Epidermal growth factor receptor 2.
ICI	Immune checkpoint inhibitor.
MIBC	Muscle-invasive bladder cancer.
NMIBC	Non-muscle-invasive BC.
PDT	Photodynamic therapy.
PIT	Photoimmunotherapy.
PPR	Pattern recognition receptor.
PS	Photosensitizer.
PSMA	Prostate-specific membrane antigen.
ROS	Reactive oxygen species.
Sac	Sacituzumab.
TF	Tissue factor.
TROP-2	Trophoblast cell-surface antigen 2.
