# Supplementary material for: Exploring EGFR, Nectin-4, and TROP-2 as Therapeutic Targets for Bladder Cancer Photoimmunotherapy
Source: Molecules. 2025 Dec 17;30(24):4802. doi: 10.3390/molecules30244802 (PMC12736122; doi:10.3390/molecules30244802)
Supplement: Supplementary file 1 [file molecules-30-04802-s001.zip › molecules-3983453-supplementary.pdf]

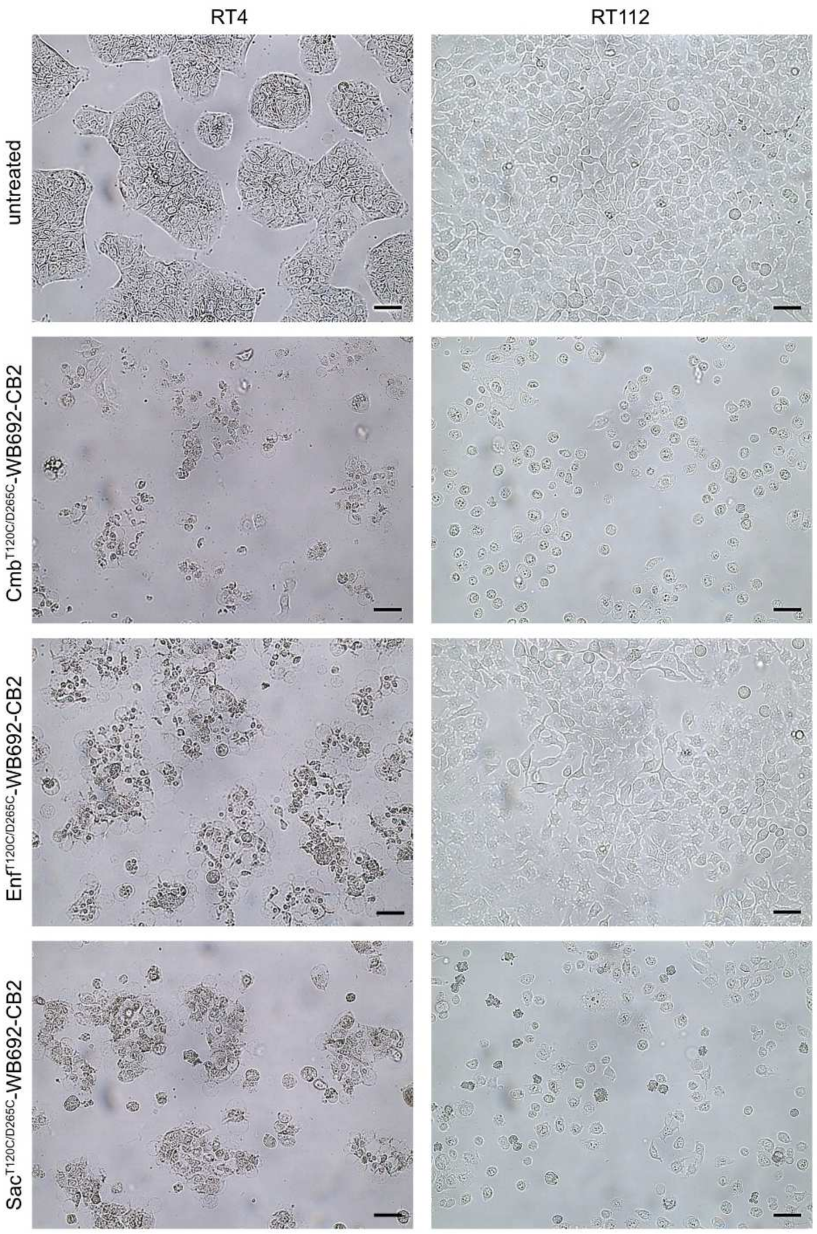

Supplementary Figure S1. Morphological changes of RT4 and RT112 bladder cancer cells following PIT. RT4 and RT112 cells were incubated with 10 μg/mL conjugates. Following incubation, cells treated with $Cmb^{T120C/D265C}$-WB692-CB2 and $Enf^{T120C/D265C}$-WB692-CB2 were irradiated with 64 J/cm², while cells treated with $Sac^{T120C/D265C}$-WB692-CB2 were irradiated with 16 J/cm². Untreated controls received no irradiation. Microscopy images were acquired 24 h after irradiation. Scale bar: 50 μm.
